# Management of Cleft Palate in Puppies Using A Temporary Prosthesis: A Report of Three Cases

**DOI:** 10.3390/vetsci5030061

**Published:** 2018-06-24

**Authors:** Theresa Conze, Isabelle Ritz, Rainer Hospes, Axel Wehrend

**Affiliations:** 1 Clinic for Obstetrics, Gynecology and Andrology of Large and Small Animals with Ambulatory Service, Faculty of Veterinary Medicine, Justus-Liebig-University, 35392 Giessen, Germany; rainer.hospes@vetmed.uni-giessen.de (R.H.); axel.wehrend@vetmed.uni-giessen.de (A.W.); 2 Department of Veterinary Clinical Science, Small Animal Clinic, Surgery, Justus-Liebig-University, 35392 Giessen, Germany; mail@isabelle.ritz.de

**Keywords:** cleft palate, palatoschisis, puppy, temporary prosthesis

## Abstract

Cleft palate in dogs is a congenital defect that mostly leads to euthanasia of the affected puppy. If an attempt is made to raise the puppy, it is generally fed via an orogastric tube. Here, we describe the management of cleft palate in three puppies (two Boxers, one Collie) using a customised temporary prosthesis, which allowed the puppies to be bottle-fed and successfully raised by their owners (Cases 2 and 3) and the author (Case 1). The temporary palatal prosthesis was manufactured from a mouthguard intended for human children, which is made of thermoplastic silicone. The preparation procedure was simple and cost-effective. All puppies underwent corrective surgery at 5–6 months of age. After surgery, one of the Boxer puppies showed mandibular mesioclusion, while the other two showed no aberrations. All puppies gained the same amount of weight as their littermates, although the weight gain of the two Boxers was slower than that of their littermates. In summary, this case report describes an easy and effective way to raise puppies with cleft palate until corrective surgery can be performed.

## 1. Introduction

Palatoschisis, also known as cleft palate (CP), is a craniofacial defect characterised by an abnormal communication between the oral and nasal cavities and is one of the most common craniofacial defects in dogs [1,2,3]. Because of this abnormal connection, suckling is impaired and the risk of aspiration pneumonia is high [4]. The only successful treatment for CP is corrective surgery, which is best performed once maxillofacial growth has slowed down or ceased completely [5]; Fiani et al. [5] recommend an age of about 4–6 months [5]. If corrective surgery is performed earlier than the recommended age, there is a high risk of dehiscence and maxillary growth delay [5]. However, raising puppies until the appropriate age for corrective surgery is challenging, and most puppies are euthanised because of the costs and intensive care required. Neonates that are raised until an age appropriate for surgery are mostly fed by an orogastric tube [6].

In the present report, we describe the successful management of CP using a customised temporary prosthesis in three puppies, and demonstrate an easy and affordable way to raise puppies with CP until corrective surgery can be performed.

## 2. Case Presentation

### 2.1. Cases

Case 1 involved a 3-day-old male Boxer puppy that presented at the Clinic for Obstetrics, Gynecology and Andrology of Large and Small Animals with Ambulatory Service of the Justus-Liebig University in Germany. The owner reported that the puppy was very active and continuously suckling. Nonetheless, he did not gain weight, and the owner frequently observed nasal discharge after suckling.

**Figure 1:**
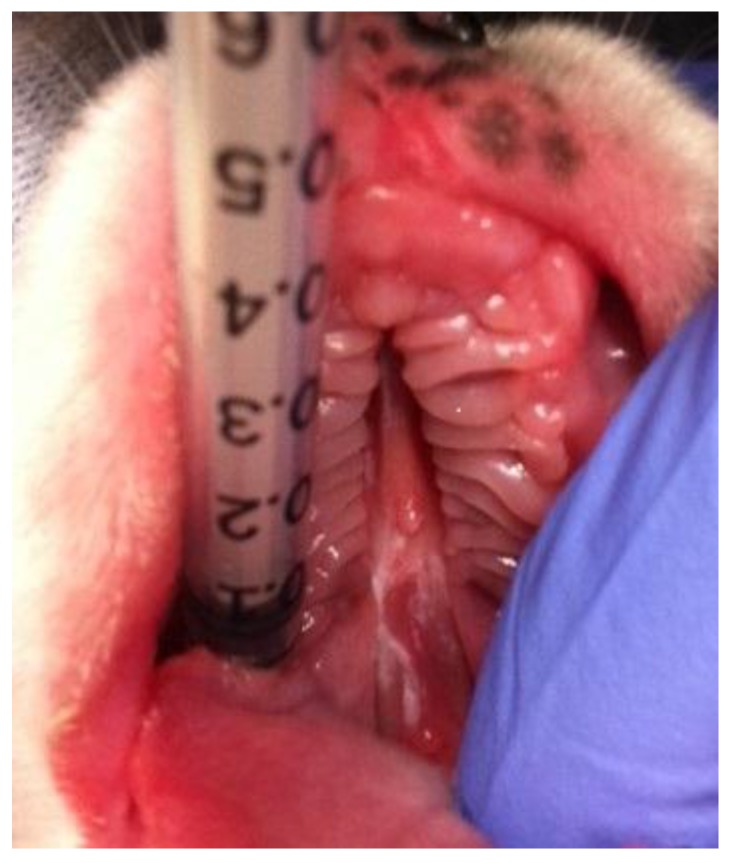
Cleft palate in a 3-day-old purebred male Boxer puppy (Case 1).

On examination, the puppy exhibited a good general condition and was very lively. Intraoral examination revealed CP (Figure 1). The CP was characterised by a midline defect in the hard palate and a caudally divergent defect in the soft palate. The defect was moderately wide, and no asymmetrical facial growth could be detected. CP was classified as “- - H S H - -“ according to the LAHSAL classification [7]. No other congenital diseases were detected, and the puppy showed no signs of pneumonia. When milk replacer (Babydog Milk^®^, Royal Canin, Köln, Germany) was offered, the puppy suckled immediately. Although he showed nasal discharge and occasional sneezing, he demonstrated a good appetite. He required a long time to consume the amount of milk necessary for gaining body weight. Because of his good general condition, an attempt at raising him was made and the puppy was assigned to the clinic.

Case 2 involved a female Boxer that presented with a congenital palatal defect on the day of birth. The puppy was diagnosed with cleft lip and palate (CLP), or cheilognathopalatoschisis, which was classified as “- - H S H A L” according to the LAHSAL classification, which is a system for classifying clefts [7]. The defect was narrow in both the hard and soft palate, measuring between 1–2 mm in width. No facial growth abnormalities were observed. The puppy showed a normal birth weight, was lively, and showed no other deformities during examination. 

Case 3 involved a male Collie that presented with mild bronchopneumonia, which was diagnosed based on a clinical examination, a complete blood count, and an ultrasound of the lungs. This puppy presented at the age of 12 days. The puppy was found to have a continuous hard and soft palate defect, classified as “- - H S H - -“ according to the LAHSAL classification [7]. The defect was narrow, and no facial growth abnormalities were seen. The owners of both puppies were willing to attempt raising them.

### 2.2. Customisation of A Palatal Prosthesis

**Figure 2:**
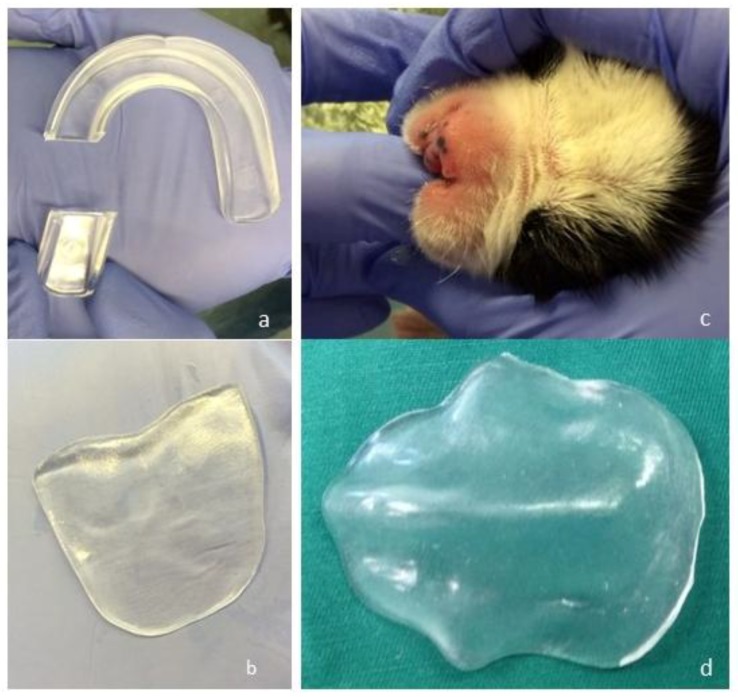
A small portion of the Wilson Single Density Youth Mouthguard (Wilson Single Density Youth Mouthguard, Wilson, Chicago, IL, USA) is cut off (**a**). A plate matching the size of the upper jaw is crafted by repeated heating, softening, and reforming the thermoplastic silicone (**b**). The warm silicone plate is pressed against the upper jaw of the puppy (**c**). The customised temporary palatal prosthesis prepared for puppies with cleft palate from the Wilson Single Density Youth Mouthguard for human children (**d**).

A temporary prosthesis was crafted from a mouthguard intended for human children (Wilson Single Density Youth Mouthguard; Wilson, Chicago, IL, USA), which is made of thermoplastic silicone and can be reformed by heating in hot water. About 1–2 cm of the mouthguard was cut off (Figure 2a) and placed in a cup with boiling water. After a few seconds, the material softened and the silicone could be reformed for about 30–60 seconds. Thus, a plate with a height of approximately 0.3 cm was prepared. During the crafting procedure, the mouthguard was heated and softened several times. After hardening, the edges of the plate were cut off until a size matching that of the upper jaw of the puppy was obtained (Figure 2b). When the optimal size and thickness (approximately 4 cm long, 2 cm wide, 0.3 cm thick) were achieved, the silicone plate was pressed against the upper jaw of the puppy (Figure 2c) to produce a prosthesis that was adjusted to the maxilla (Figure 2d). No anaesthesia was required during this procedure. The prosthesis was only inserted in the mouth during bottle feeding and did not require fixation. The puppies tolerated the prosthesis well and showed rapid and normal milk intake. Although small amounts of nasal discharge were occasionally observed after drinking, sneezing and coughing were never observed, and there were no signs of aspiration. After 2 weeks, a new prosthesis had to be customised, as described above, because of physiological growth of the puppy. At 6 weeks of age, the owners began mixing the puppy milk (Babydog Milk^®^, Royal Canin, Köln, Germany) with wet dog food (Starter Mousse^®^, Royal Canin, Köln, Germany), and at 2.5 months of age, they changed the feed to commercial dry dog food (Puppy Large Breed^®^, Eukanuba, Coevorden, Netherlands), which the puppies could eat without the prosthesis. The puppies showed no difficulty in drinking water. 

### 2.3. Further Therapies

All puppies were treated with frequent inhalation of ambroxol during the first 2 weeks (Mucosolvan^®^, 6 mg per inhalation, Boehringer Ingelheim, Ingelheim am Rhein) to clear the upper respiratory tract. The puppy in Case 3 received amoxicillin–clavulanic acid for 10 days (Synulox^®^, 12.5 mg/kg body weight, orally, twice a day, Zoetis, Berlin, Germany) for the management of bronchopneumonia.

### 2.4. Development

The puppy in Case 1 was raised by one of the authors in isolation from its littermates, while the other two puppies were raised along with their littermates by their respective owners. Both of them suckled from their mothers without the prosthesis; however, there was no effective milk intake. All puppies were fed by bottle with the prosthesis in place. The puppy in Case 2 showed signs of pneumonia at approximately 6 weeks of age, but recovered and developed well after the treatment with amoxicillin–clavulanic acid (Synulox^®^, 12.5 mg/kg body weight, orally, twice a day, Zoetis, Berlin, Germany). Although the puppies showed good food intake, the two Boxer puppies exhibited slower body weight gain than their littermates, while the Collie developed at the same rate as his littermates.

### 2.5. Outcomes and Follow-up

**Figure 3:**
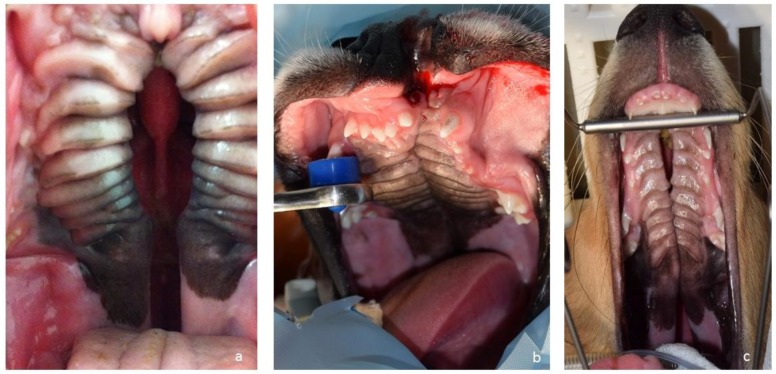
Cleft palate/cleft lip and palate on the day of corrective surgery in Case 1 (**a**), Case 2 (**b**) and Case 3 (**c**).

**Figure 4:**
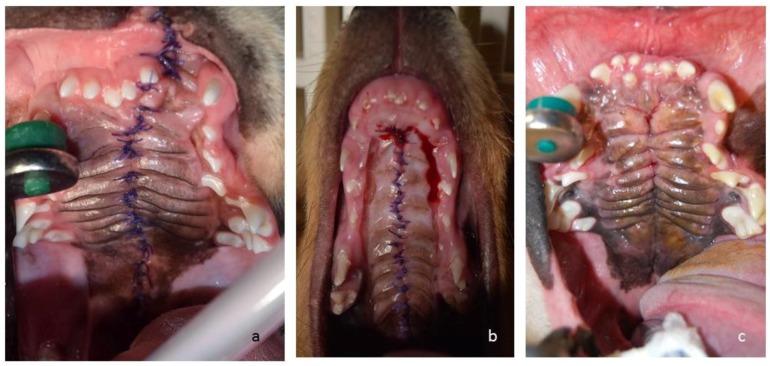
Intraoral images of Case 2 (**a**) and Case 3 (**b**) 3 days after surgery, and Case 1 (**c**) 2 months after surgery. Cases 2 and 3 did not require any further surgery. Case 1 still showed some fistulas 2 months after surgery, which were successfully closed.

**Figure 5:**
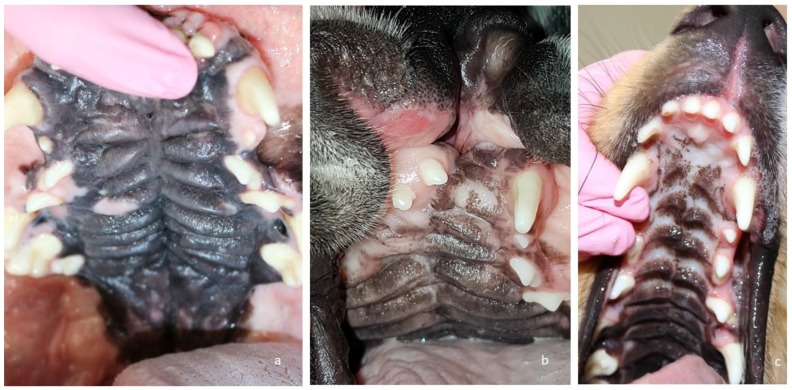
Intraoral images of Case 1 (**a**) 4 years and Case 2 (**b**) and Case 3 (**c**) 2 years after surgery.

Between 5–6 months of age, all three puppies (Figure 3) underwent corrective surgery. Pre-operatively, a complete blood count and a blood chemistry test were performed on all dogs. All values were within normal range. For corrective surgery, the medially repositioned double-flap technique (von Langenbeck technique) was used for the hard palate [8] and the double-layer appositional technique was used for the soft palate. The surgical wounds healed well, with normal symmetry between the right and left sides of the maxilla (Figure 4). The puppy in Case 1 developed mandibular mesioclusion after surgery. The cleft lip wound in the puppy in Case 2 opened up after surgery, but the owner decided against further corrective surgery because there was no functional constraint. All puppies achieved the same body size and weight as their littermates. At the time of writing this article, the dogs were aged 2–4 years. All dogs were healthy and showed no problems associated with their congenital defects (Figure 5). The cleft lip persisted in the puppy in Case 2, but this represents a cosmetic defect only.

## 3. Discussion

Although CP is one of the most common congenital craniofacial defects in dogs, most puppies are euthanised or die because of aspiration pneumonia. Corrective surgery can be performed at an age of about 4–6 months [5]. Until the puppy is old enough for surgery, nutrition has to be ensured, and the risk of aspiration pneumonia has to be minimised. In children, a feeding obturator is commonly provided until corrective surgery is performed [9,10]. The preparation of prosthodontic obturators after corrective surgery for congenital as well as acquired cleft palates in dogs and cats has been previously described [2,11,12]. Martinez-Sanz et al. [4] described the preparation of customised feeding teats using a thermovacuum-forming machine under anaesthesia for the management and raising of puppies with CP before surgery. Although they described an effective way to raise puppies with CP, the procedure for manufacturing the teats and palatal prosthesis is complex and requires special equipment as well as anaesthesia. In the cases presented here, the preparation procedure for the palatal prosthesis was inexpensive, simple, and effective; moreover, no anaesthesia was needed, all of which are important factors in the willingness of owners to raise a puppy with CP. Although a palatal prosthesis needs to be prepared several times as the puppy grows, only one mouthguard (Wilson Single Density Youth Mouthguard) could supply all the material necessary for all the prostheses. Furthermore, the owners could replicate the manufacturing procedure after observing it once, although regular supervision by a veterinarian is recommended. This approach allows the puppy to grow up in a normal social environment. This approach also makes bottle feeding and suckling possible (which are important for normal craniofacial growth [13]) with minimal risk of milk aspiration and subsequent pneumonia. This prosthesis allows for the normal raising of such puppies until most of the maxillofacial growth is complete, preventing maxillofacial deformities and inhibiting maxillary growth that can occur because of palatoplastics [8,14,15]. Although the puppy in Case 1 developed mandibular mesioclusion after surgery, this can be attributed to the breed and was not considered a consequence of the surgery. The optimal time and technique for surgery should be chosen according to the size of the cleft palate [7,16,17,18,19]. Although there are various reasons for the development of congenital palatoschisis, there is a genetic predisposition [20,21,22], and castration of the animal is strongly advised.

## 4. Conclusions

In summary, we described the successful management of puppies with CP using a temporary customised prosthesis, which allowed them to be bottle-fed and successfully raised until corrective surgery could be performed. The findings from this report show that a puppy with CP can be raised with little effort and at low cost, thus encouraging owners to seek appropriate treatment for such puppies instead of considering euthanasia.
